# Approximate Observation Weighted *ℓ*_2/3_ SAR Imaging under Compressed Sensing

**DOI:** 10.3390/s24196418

**Published:** 2024-10-03

**Authors:** Guangtao Li, Dongjin Xin, Weixin Li, Lei Yang, Dong Wang, Yongkang Zhou

**Affiliations:** 1 School of Information Science and Engineering, University of Jinan, Jinan 250022, China; guangtaoli@stu.ujn.edu.cn (G.L.); ise_liwx@ujn.edu.cn (W.L.); ise_yangl@ujn.edu.cn (L.Y.); ise_wangdong@ujn.edu.cn (D.W.); 202321200874@stu.ujn.edu.cn (Y.Z.); 2 Shandong Provincial Key Laboratory of Ubiquitous Intelligent Computing, Jinan 250022, China

**Keywords:** sparse recovery, approximated observation, weighted *ℓ*_2/3_ regularization

## Abstract

Compressed Sensing SAR Imaging is based on an accurate observation matrix. As the observed scene enlarges, the resource consumption of the method increases exponentially. In this paper, we propose a weighted *ℓ*_2/3_-norm regularization SAR imaging method based on approximate observation. Initially, to address the issues brought by the precise observation model, we employ an approximate observation operator based on the Chirp Scaling Algorithm as a substitute. Existing approximate observation models typically utilize *ℓ*_*q*_(*q* = 1, 1/2)-norm regularization for sparse constraints in imaging. However, these models are not sufficiently effective in terms of sparsity and imaging detail. Finally, to overcome the aforementioned issues, we apply *ℓ*_2/3_ regularization, which aligns with the natural image gradient distribution, and further constrain it using a weighted matrix. This method enhances the sparsity of the algorithm and balances the detail insufficiency caused by the penalty term. Experimental results demonstrate the excellent performance of the proposed method.

## 1. Introduction

Synthetic Aperture Radar (SAR) is a remote sensing radar capable of acquiring high-resolution images of the Earth’s surface, irrespective of weather conditions and time of day [1]. Traditional SAR imaging methods require the sampling rate to adhere to the Nyquist sampling theorem. If further resolution enhancement is desired, the sampling rate must be increased, which imposes stringent demands on hardware and data storage. Compressed Sensing (CS), introduced by Donoho et al. [2], has been extensively deployed in diverse domains over the past decades, including wireless communication [3], radar signal processing [4], and image restoration [5]. The CS theory uses the sparsity of signals for compressive sampling and employs sparse reconstruction algorithms for signal recovery. The signal bandwidth is no longer tied to the sampling rate of the reconstructed image, thereby reducing the sampling rate and alleviating hardware constraints.

Early CS-SAR imaging was based on an accurate observation matrix. Ref. [6] applied range compression and range cell migration correction to the original SAR echo data, constructed an azimuth observation matrix for each range gate, and reconstructed the image using sparse reconstruction methods. Zeng et al. [7] proposed that SAR echoes from small scenes could benefit from *ℓ*_1/2_ regularization constraint optimizations during reconstruction. For large scenes, the multi-segment CS method segments the scene along the range dimension, performs sparse reconstruction on the segmented sub-scenes, and finally synthesizes the entire image [8].

The aforementioned methods are based on traditional CS methods and require data processing after range compression and range cell migration correction. When the scene scale increases, the observation matrix also enlarges, which not only fails to reduce the system’s complexity and sampling rate but also increases the complexity of signal processing. Ref. [9] proposed an approximate observation SAR imaging model, which employed *ℓ*_1_ regularization for sparse constraints. This method effectively reduces the resource consumption and complexity of traditional CS-SAR imaging methods. In SAR moving target imaging, to address the challenges of mixed echoes from moving and static targets and the lack of prior information, An et al. [10] incorporated the inverse operator of an imaging algorithm into a joint observation model for both moving and static targets. The authors employed a joint optimization imaging approach based on Particle Swarm Optimization (PSO) and the Alternating Direction Method of Multipliers (ADMM). Xu et al. [11] proposed an SAR imaging method based on *ℓ*_1/2_ regularization, which achieved sparse reconstruction by constructing an imaging operator. Regularization methods can be solved using gradient descent algorithms [12], iterative thresholding algorithms [13], and so on. Tensor-based and low-rank methods have been widely applied in MIMO radar imaging and SAR imaging. For integrated sensing and communication (ISAC) and massive MIMO scenarios, Zhang et al. [14] proposed a unified tensor approach for channel and target parameter estimation. This method provided a unified framework for channel and target estimation, improving computational efficiency and reducing complexity, making it more suitable for large-scale MIMO radar imaging applications. Based on a unified framework, ref. [15] combined channel estimation and target sensing using a shared training pattern, integrating target sensing into the channel estimation stage. This approach addressed the computational burden and training overhead associated with utilizing a large number of antennas in terahertz ISAC systems, as well as the difficulty of simultaneously estimating channel and target parameters using conventional methods, thereby extending the applicability of MIMO radar imaging scenarios. Ref. [16] modeled the video SAR imaging problem as a joint recovery of low-rank and sparse tensors and employed the tensor ADMM to reconstruct high-quality SAR images from a reduced number of samples.

In the regularization constraint models, the smaller the value of *q*, the more the *ℓ_q_* regularization term promotes the sparsity of the solution. When q=0, this regularization becomes an NP-hard problem, typically approximated using greedy algorithms such as orthogonal matching pursuit (OMP) [17], regularized orthogonal matching pursuit (ROMP) [18], and iterative soft thresholding algorithms (ISTAs) [19]. Directly solving with greedy algorithms often consumes substantial resources. To address the NP-hard problem and achieve appropriate sparsity and performance, the *ℓ_q_* norm (0< *q* ≤1) is chosen to replace the *ℓ*_0_ norm. Common values for *q* are 1/2 and 1. However, the gradient distribution of natural images follows a heavy-tailed super-Laplacian distribution [20] $F\left(X\right)\propto e^{-k|X{|}^{q}}$, where *K* denotes sparsity and 0.5≤ *q* ≤0.8. Based on this prior information, selecting *ℓ*_2/3_ regularization is of great significance for SAR imaging in natural scenes.

In this paper, we present an SAR imaging method using weighted *ℓ*_2/3_ regularization based on approximate observations. Initially, we build an observation operator using the Chirp Scaling Algorithm, suited for fast imaging in large areas. We then apply weighted *ℓ*_2/3_ regularization, aligning with the natural image gradient distribution. This step improves the method’s sparsity, balances the penalty term’s intensity, and preserves image edge details. Lastly, we propose a weighted iterative threshold algorithm for this method. Through experiments, we demonstrate the method’s superior reconstruction performance.

## 2. Echo Signal Model for Stripmap Radar Imaging

**Figure 1:**
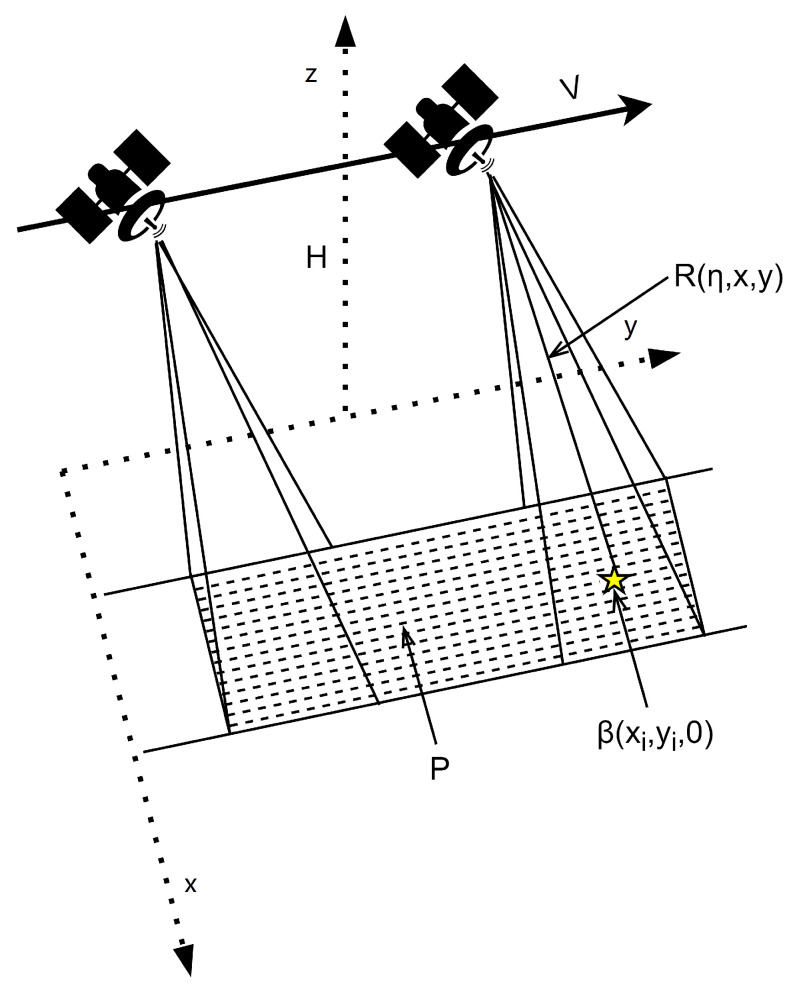
Stripmap radar imaging model.

The geometric structure of the stripmap radar imaging is illustrated in Figure 1. Let x,y,z denote the Cartesian coordinate system. The radar platform moves with a velocity *v* along the *y*-axis, transmitting electromagnetic waves towards the observation area *P* at a certain depression angle. The slant range between the radar and the point target $T_{i}\left(x,y,0\right)$ is R(η,x,y), where η represents the azimuth time, and *x* and *y* are the coordinates of the point target. The altitude of the radar platform is *H*.

Assume that the radar transmits a linear frequency-modulated (LFM) signal denoted as

(1) $$q\left(t\right)=rect\left(t/T\right)\exp\left\{j\pi at^{2}\right\}$$

where *t* is the fast time variable, *a* is the chirp rate, *T* is the pulse width, and rect(·) is the window function. Assuming the scene contains only a single point target β, the demodulated baseband echo signal is as

(2) $$s\left(t,\eta \right)=\underset{P}{\;\int \int \;}g\left(x,y\right)q\left[t-2\frac{R(\eta ,x,y)}{c}\;\cdot \;\exp\left[-j\frac{4\pi f_{c}R\left(\eta ,x,y\right)}{c}\right]dxdy\right]$$

where *P* denotes the radar antenna’s illuminated area, *g* represents the scattering coefficient of a target point in the scene, $f_{c}$ is the carrier frequency, and *c* is the speed of light. To further process the echo signal, we discretize the scene and the continuous-time signal, resulting in the following echo data:

(3) $$s\left(\varrho ,\zeta \right)=\sum_{l=1}^{L}g\left(l\right)p\left[t\left(\zeta \right)-\frac{2R(\varrho ,l)}{c}\right]\;\cdot \;\exp\left[-j\frac{4\pi f_{c}R\left(\varrho ,l\right)}{c}\right]$$

where l=1,2,3,···,L represents the *l*-th target in the imaging scene, and *L* is the total number of targets after discretizing the scene. ϱ represents the ϱ-th observation position after discretizing slow time, and ζ represents the ζ-th fast time sampling point after discretizing fast time.

## 3. Proposed Methods

### 3.1. SAR Imaging Model Based on Approximate Observation

The Chirp Scaling is a widely utilized matched-filtering algorithm for SAR imaging [21], characterized by a relatively simple framework that comprises phase compensation and Fast Fourier Transform (FFT). The imaging operator based on Chirp Scaling can be represented as

(4) $$U\left(Y\right)=F_{a}^{-1}\left(F_{a}Y⊙P_{sc}F_{r}⊙P_{rc}F_{r}^{-1}⊙P_{ac}\right)$$

where Y denotes the original two-dimensional SAR echo data. $F_{r}$ and $F_{a}$, respectively, represent the Fourier transforms in the range and azimuth directions, and $F_{r}^{-1}$ and $F_{a}^{-1}$, respectively, denote the inverse Fourier transforms in the range and azimuth directions. $P_{\mathrm{sc}}$, $P_{\mathrm{ac}}$, and $P_{\mathrm{rc}}$, respectively, signify the decoupling operator, azimuth compression and phase correction operator, and range compression and range cell migration correction operator [9]. ⊙ represents the Hadamard product. $U\in C^{M\;\times \;N}$ is the imaging operator, *M* indicates the number of sampling points in the azimuth direction, and *N* represents the number of sampling points in the range direction. Due to the imaging operators being a series of linear matrix multiplications, the approximate observation operator can be derived by calculating its inverse process:

(5) $$I\left(X\right)=F_{a}^{-1}\left(F_{a}X⊙P_{ac}^{*}F_{r}⊙P_{rc}^{*}F_{r}^{-1}⊙P_{sc}^{*}\right)$$

where X represents the two dimensional complex image data of the observed scene, and ${\left(\;\cdot \;\right)}^{*}$ denotes the matrix conjugate operation. $I\in C^{M\times N}$ is the approximate observation operator. The relationship between the imaging operator U and the approximate observation operator I is as follows:

(6) $$I=U^{-1}=U^{*}$$

The Chirp Scaling SAR imaging model based on approximate observation can be represented as

(7) $$\underset{X}{min}\left\{{\left\|Y-I\left(X\right)\right\|}_{F}^{2}+\lambda {\left\|X\right\|}_{q}^{q}\right\}$$

where ${\left\|\;\cdot \;\right\|}_{F}$ denotes the Frobenius norm of the matrix, ${\left\|\;\cdot \;\right\|}_{q}^{q}$ (0≤ *q* ≤1) represents the regularization term, and λ>0 is the regularization parameter, which plays a role in balancing the reconstruction accuracy and sparsity.

### 3.2. Weighted ℓ_2/3_ Regularization Model

For *ℓ*_0_ regularization, each non-zero element is penalized equally within the objective function. Conversely, with relaxed *ℓ_q_* regularization, elements of varying magnitudes are penalized differently within the objective function. To approximate the *ℓ*_0_ norm as closely as possible and address the unequal penalty on non-zero coefficients, we propose a weighted *ℓ*_2/3_ regularization formulation based on approximate observation.

(8) $$\underset{X}{min}\left\{{\left\|Y-I\left(X\right)\right\|}_{F}^{2}+\lambda {\left\|W⊙X\right\|}_{2/3}^{2/3}\right\}$$

where $W\in R^{M\;\times \;N}$ denotes the weighting matrix. ⊙ represents the Hadamard product. In terms of weight setting, to better approximate *ℓ*_0_ regularization with *ℓ*_2/3_ regularization, the weights in the weighting matrix should be inversely proportional to the magnitudes of the true signal. This approach effectively balances the penalties among different magnitudes while further enhancing the sparsity constraint capability.

### 3.3. Weighted Iterative Thresholding Algorithm

To solve the aforementioned model, we propose a weighted iterative thresholding algorithm. To obtain a set of effective weights without knowing the estimate of X, the weights are initially set to W=1 in the first iteration. Based on the theoretical analysis above, the expression for the weighting matrix is given by

(9) $$w_{m,n}=\frac{1}{|x_{m,n}|+\epsilon }$$

where, $w_{m,n}$ and $x_{m,n}$ are the elements of *m* rows and *n* columns in W and X, and the constant ϵ>0 serves to prevent the denominator from being zero. The construction of weights via an iterative algorithm allows for continual updating based on the estimated values of X. The closer the estimated X is to the true value, the more the weighted *ℓ*_2/3_ regularization approximates *ℓ*_0_ regularization. The iterative weighting algorithm identifies large magnitude elements during the iteration process and reduces their influence, making it easier to detect small-magnitude non-zero elements. The expression for the iterative thresholding algorithm based on the approximately observed weighted *ℓ*_2/3_ is given by

(10) $$X^{i+1}=H_{W,\lambda ,\mu ,2/3}\left[X^{i}+\mu U\left(Y-I\left(X^{i}\right)\right)\right]$$

where μ is the iterative step size. $H_{W,\lambda ,\mu ,2/3}$ denotes the thresholding operator [20]

(11) $$H_{W,\lambda ,\mu ,2/3}\left(z\right)=\left[\begin{array}{cccc} {\bar{h}}_{w_{1,1}\lambda ,\mu }\left(z_{1,1}\right) & {\bar{h}}_{w_{1,2}\lambda ,\mu }\left(z_{1,2}\right) & \cdots & {\bar{h}}_{w_{1,n}\lambda ,\mu }\left(z_{1,n}\right)\\ {\bar{h}}_{w_{2,1}\lambda ,\mu }\left(z_{2,1}\right) & {\bar{h}}_{w_{2,2}\lambda ,\mu }\left(z_{2,2}\right) & \cdots & {\bar{h}}_{w_{2,n}\lambda ,\mu }\left(z_{2,n}\right)\\ \vdots & \vdots & \ddots & \vdots \\ {\bar{h}}_{w_{m,1}\lambda ,\mu }\left(z_{m,1}\right) & {\bar{h}}_{w_{m,2}\lambda ,\mu }\left(z_{m,2}\right) & \cdots & {\bar{h}}_{w_{m,n}\lambda ,\mu }\left(z_{m,n}\right) \end{array}\right]$$

within this context, $X^{i}+\mu U\left(Y-I\left(X\right)\right)=z$, and the expression for ${\bar{h}}_{w_{m,n}\lambda ,\mu }\left(z_{m,n}\right)$ is as follows:

(12) $${\bar{h}}_{w_{m,n}\lambda ,\mu }\left(z_{m,n}\right)=\left\{\begin{array}{cc} h_{w_{m,n}\lambda ,\mu }\left(z_{m,n}\right), & |z_{m,n}|>\frac{\sqrt[{4}]{48}}{3}{\left(w_{m,n}\lambda \mu \right)}^{\frac{3}{4}}\\ 0, & otherwise \end{array}\right.$$

where $h_{w_{m,n}\lambda ,\mu }\left(z_{m,n}\right)$ is defined by

(13) $$\begin{array}{c} \;\;\;\;\;\;h_{w_{m,n}\lambda ,\mu }\left(z_{m,n}\right)\;=\;sgn\left(z_{m,n}\right)\;{\left(\begin{array}{c} \frac{\sqrt{\varphi (z_{m,n})}+\sqrt{2\sqrt{\varphi^{2}\left(z_{m,n}\right)\;-\;\frac{4}{3}w_{m,n}\mu \lambda }\;-\;\varphi \left(z_{m,n}\right)}}{2} \end{array}\right)}^{3}\;\;\;\;\;\;\; \end{array}$$

where $\varphi (z_{m,n})$ is defined by

(14) $$\;\;\;\;\;\;\;\varphi \left(z_{m,n}\right)=\frac{4}{3}{\left(w_{m,n}\mu \lambda \right)}^{\frac{1}{2}}\cosh\left(\frac{1}{3}\mathrm{arcosh}\left(\frac{27}{16}{\left(w_{m,n}\mu \lambda \right)}^{-\frac{3}{2}}z_{m,n}^{2}\right)\right)\;\;\;\;\;$$

From the above equation, in each iteration, gradient estimation is performed using the information at the current point, followed by weighted threshold shrinkage (Algorithm 1).
**Algorithm 1** Weighted *ℓ*_2/3_ Iterative Thresholding Algorithm**Input:** SAR raw echoes Y, approximated observation operator I and imaging operator U,    weighting matrix W Initial $X^{0}=0$
μ and Maximum Number of Iterations $I_{\max}$
**Output:** The recovery image X
  1:  for $i=0:I_{max}$
  2:    Residue: $Re^{i}=Y-I\left(X^{i}\right)$
  3:    Matched Filter: $\Delta X^{i}=U\left(Re^{i}\right)$
  4:    Gradient Descent: $B_{\mu }\left(X^{i}\right)=X^{i}+\mu \Delta X^{i}$
  5:    Threshold Shrinkage [20]: $X^{i}=H_{W_{m,n}\mu ,\lambda }\left(B_{\mu }\left(X^{i}\right)\right)$
  6:  end for


### 3.4. Parameter Setting

In the algorithmic workflow, μ and λ need to be specified. The iterative step size μ controls the convergence speed of the iterative thresholding algorithm, and this parameter must satisfy $\mu^{-1}\;>\left\|\;I\left(U\left(\;\cdot \;\right)\right)\;\right\|$. λ is the regularization parameter that balances the reconstruction accuracy and sparsity of the objective function. For the $K_{0}$ sparse problem, the regularization parameter can be set as

(15) $$\lambda =\frac{2\left|B_{\mu }{\left(X^{i}\right)}_{e,r}\right|}{w_{e,r}}$$

where *e* and *r* are the coordinates of the $\left(K_{0}+1\right)$-th largest element in matrix $B_{\mu }\left(X^{i}\right)$ by magnitude.

## 4. Simulations and Applications

In this section, we validate the effectiveness of the proposed algorithm through simulations and real-world applications. The simulation data are generated using a model of five ideal point targets. For the applications, we utilize real datasets collected from the RADARSAT-1 and Gaofen-3 satellites over a harbor area. This scene includes various types of ships and represents a typical sparse scenario. Initially, we conduct a series of simulation comparisons, primarily focusing on the matched-filter-based Chirp Scaling Algorithm (CSA), as well as iterative algorithms with *ℓ*_1_(*ℓ*_1_-ITA), *ℓ*_1/2_(*ℓ*_1/2_-ITA), and *ℓ*_2/3_(*ℓ*_2/3_-ITA) thresholding based on Compressed Sensing regularization. The reconstruction performance is evaluated using metrics such as MSE, ENL, RaRes, and ENY. Finally, the proposed method is applied to real datasets to further demonstrate its superiority. Imaging employs the SAR stripmap mode, and all simulation experiments are conducted in the MATLAB R2022b software environment, with the computer configuration being an Intel Core I7-12700 and 16 GB of RAM.

1. ENL: Equivalent number of looks is an indicator used to measure the smoothness of homogeneous regions in an image. Generally, a higher ENL value indicates a greater degree of speckle noise suppression by the imaging method [22].
2. RaRes: Radiometric resolution can be used to evaluate the ability of an SAR imaging method to distinguish between adjacent scattering coefficients. A higher value indicates a lower ability to resolve adjacent targets [23].
3. ENY: Entropy is an index of the complexity and randomness within image content. In SAR imaging, a lower entropy value denotes reduced random noise and, consequently, enhanced interpretability and clarity of the imaged information [24].
4. MSE: Mean squared error measures the deviation between estimated values and actual values. A smaller MSE value implies that the reconstructed image more closely approximates the original scene [25].

### 4.1. Simulations

**Figure 2:**
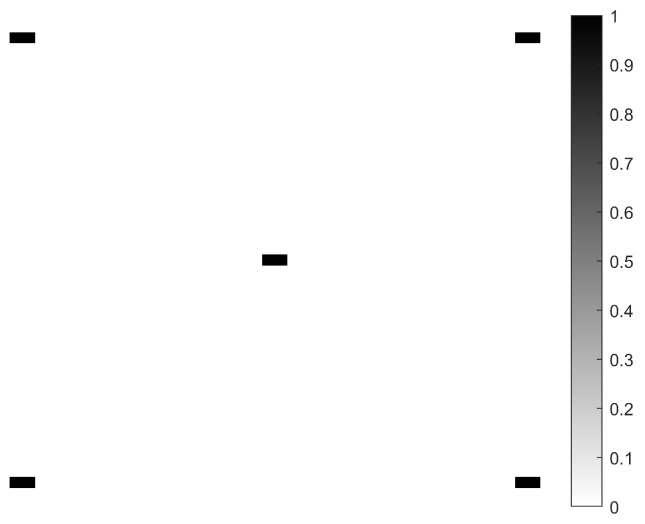
Imaging map of five point targets under the original scene.

**Figure 3:**
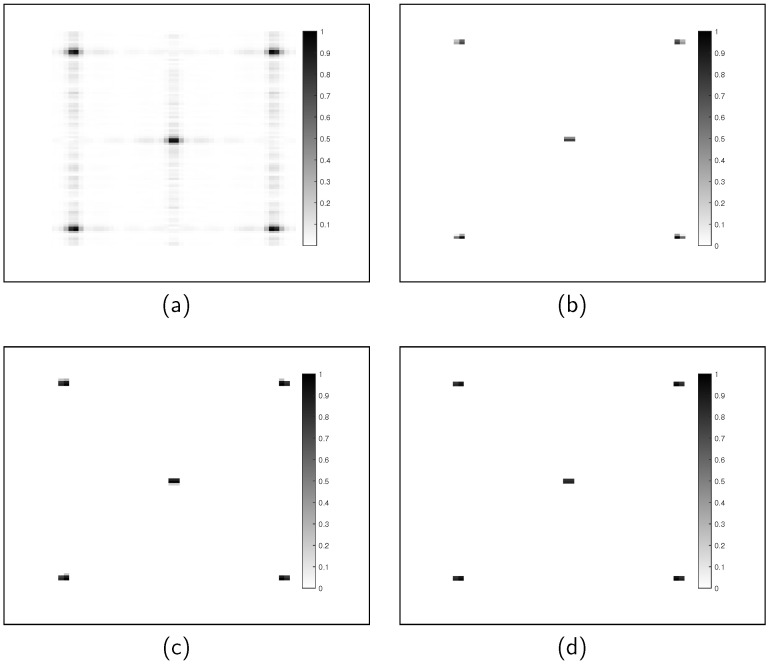
The reconstruction results of five point targets are presented under a scenario where the SNR is at 10dB and there is a 70 percent data loss in the azimuth direction. (**a**) CSA. (**b**) *ℓ*_1_-ITA. (**c**) *ℓ*_1/2_-ITA. (**d**) The proposed method.

In the simulation experiments, targets within a designated area are scanned by radar, and the slant range of each target point is computed in the time domain to generate two-dimensional raw echo data. Measurements are obtained by down-sampling the raw data. We set the azimuth sampling points to M=2048 and range sampling points to N=1024, and the two-dimensional echo data are generated using the simulation parameters listed in Table 1. The original imaging scenario of the ideal point targets is shown in Figure 2, where the scattering coefficients of the five point targets are identical. To evaluate the performance of the method, the signal-to-noise ratio (SNR) is set to 10 dB, and 70% of the azimuth data are missing. The sparsity K=10 is used for the reconstructed images of the four methods. Figure 3 presents the reconstructed images using the five methods. In Figure 3a, when the sampling rate is reduced, the CSA exhibits significant sidelobes and noise in the reconstructed image. In contrast to CSA, the *ℓ*_1_-ITA, *ℓ*_1/2_-ITA, and the proposed method mitigate most of these issues. The imaging result with *ℓ*_1_-ITA regularization, shown in Figure 3b, reveals some missing target information. The results with *ℓ*_1/2_-ITA, illustrated in Figure 3c, demonstrate that although the target images are clearly reconstructed, some sidelobes and artifacts remain. The imaging results of the proposed algorithm, as shown in Figure 3d, effectively mitigate the sidelobe issues while better preserving the edge details of the targets. Table 2 compares the performance of the four methods, with the proposed method demonstrating the best performance.

### 4.2. Applications

**Figure 4:**
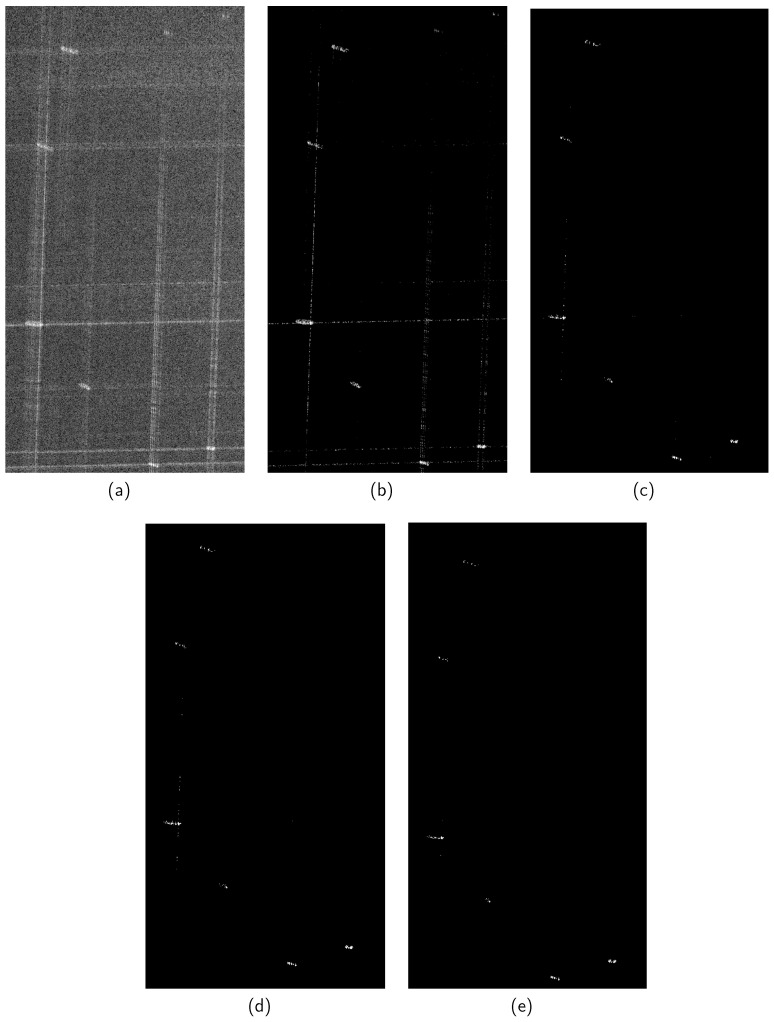
RADARSAT-1 application uses 36% data. (**a**) CSA. (**b**) *ℓ*_1_-ITA. (**c**) *ℓ*_1/2_-ITA. (**d**) *ℓ*_2/3_-ITA. (**e**) The proposed method.

RADARSAT-1, launched by the Canadian Space Agency in 1995, is a well-known SAR satellite [26]. The experimental data used in this chapter are SAR echoes from the Vancouver area, Canada, in 2002. The azimuth sampling points are set to M=4096 and the range sampling points to N=4096. For easier visualization, we crop the sparse targets from the data. The scene, containing six ships, is a typical sparse scenario. We use 36% of the data for sparse reconstruction, setting the sparsity level at K=20,000. The relevant parameters of the SAR system are shown in Table 1. Figure 4 presents the reconstruction results of four methods. CSA imaging exhibits pervasive background noise and significant sidelobe effects. *ℓ*_1_-ITA imaging eliminates most of the noise but has weaker suppression of sidelobes and artifacts. *ℓ*_1/2_-ITA imaging addresses most of these issues, showing superior overall performance, though sidelobes appear in the range direction of a single large-scale target. For applications in natural scenes, *ℓ*_2/3_ regularization is more suitable. The proposed method preserves the target edge details while isolating interference noise, resulting in better imaging quality. Table 3 compares the performance of the four methods, with the proposed method demonstrating the best performance.

**Figure 5:**
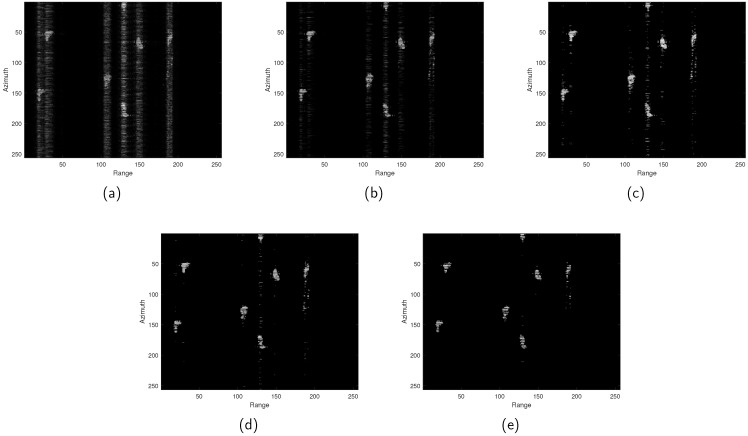
GaoFen-3 application uses 50% data. (**a**) CSA. (**b**) *ℓ*_1_-ITA. (**c**) *ℓ*_1/2_-ITA. (**d**) *ℓ*_2/3_-ITA. (**e**) The proposed method.

We conduct experiments using real-world data of port vessels acquired by the GaoFen-3 satellite [27]. In this experiment, we first apply five algorithms to reconstruct the port area, which features a typical sparse scenario with seven sparsely distributed vessels. Then, we reconstruct images using five different methods with a sparsity level of K=2000 and conduct a comparative analysis under a 50% sampling rate. We set the azimuth sampling points to M=256 and range sampling points to N=256. Some typical reconstruction results are shown in Figure 5, demonstrating performance similar to the simulation results. As shown in Figure 5a, after subsampling to 50%, CSA fails to reconstruct the image effectively, exhibiting strong sidelobes and noticeable blurriness. In contrast, other algorithms demonstrate significant improvements in these aspects. Figure 5b–d show that *ℓ*_1/2_-ITA and *ℓ*_2/3_-ITA achieve higher reconstruction accuracy compared to *ℓ*_1_-ITA. Although *ℓ*_2/3_-ITA exhibits a lower entropy value than *ℓ*_1/2_-ITA in image reconstruction, its ENL is also lower, indicating that its noise suppression capability is inferior to the latter. To further enhance the performance of *ℓ*_2/3_-ITA and improve its sparsity capability, we employ a weighted matrix. As clearly shown in Figure 5e and Table 4, the proposed method achieves higher reconstruction accuracy and the best metrics across various performance evaluations.

### 4.3. Performance Analysis

The experiments presented above demonstrate the reconstruction performance of five different methods, enabling a discussion of these methods based on the reconstructed images and evaluation metrics. The traditional imaging method based on matched filtering shows suboptimal performance, as it is constrained by the sampling theorem and cannot achieve undersampling imaging. However, its simplicity and universality make it suitable for various SAR imaging scenarios. CS SAR imaging based on an exact observation matrix is not suitable for large-scale scenes. The regularization method based on approximate observations addresses this issue and demonstrates excellent performance. In point target imaging, the *ℓ*_1_-ITA reconstruction results exhibit target loss, with lower MSE and ENL, indicating weaker stability and noise resistance. *ℓ*_1/2_-ITA improves upon *ℓ*_1_-ITA, but some sidelobes are present. The parameters indicate that its stability and sparsity have been enhanced. The proposed method, due to the influence of the weighted matrix, exhibits excellent performance in point target simulations. For satellite images, *ℓ*_2/3_-ITA achieves better reconstruction accuracy than *ℓ*_1/2_-ITA because it is more suitable for natural images. Although *ℓ*_2/3_-ITA and *ℓ*_1/2_-ITA show significant improvements over CSA and *ℓ*_1_-ITA, their reconstruction results still exhibit some residual sidelobes across all datasets. We applied a weighting matrix to the *ℓ*_2/3_ regularization method based on approximate observations. The aforementioned experiments and performance metrics indicate the feasibility and advantages of the proposed method, but it is constrained by sparse scenes. We also aim to extend the method to broader scenarios in the next step, leveraging deep learning to address its limitations.

## 5. Conclusions

This paper proposes an SAR imaging method based on approximate observation weighted *ℓ*_2/3_ regularization. Compared to traditional CS-SAR imaging, we introduce an approximate observation operator that reduces hardware demands and enables rapid imaging. By combining the weighted matrix with *ℓ*_2/3_ regularization, we address the imbalance in the penalization of elements in the regularization term. Under the influence of the weighted matrix, the method exhibits superior noise resistance, better preserving the edges and details of targets and enhancing the sparsity of the solution. Compared to other methods, under the same parameters, the proposed method achieves better high-resolution imaging in sparse scenarios.

## Figures and Tables

**Table 1:** Primary parameters of SAR system and geometry.

Parameter	Simulation	Applications
Slant range of scene center (km)	750	1016.7
Effective radar velocity (m/s)	7100	7062
Beam squint angle (rad)	0	0.06
Radar center frequency (MHz)	10,000	5300
Pulse duration (μs)	20	30
Range FM Rate (MHz/μs)	1.5	0.72135
Range Sampling Rate (MHz)	36	32.317
Azimuth Sampling Rate (Hz)	2841	1733

**Table 2:** Performance comparison of different methods on point target dataset.

Method	MSE	ENL	ENY	RARES	Imaging Time
CSA	1.01	0.16	0.54	5.40	1.20 s
*ℓ*_1_-ITA	1.32 × 10^−2^	7.31	2.10 × 10^−4^	1.36	3.38 s
*ℓ*_1/2_-ITA	1.18 × 10^−2^	11.77	2.18 × 10^−4^	1.11	2.39 s
The proposed method	7.60 × 10^−3^	26.46	1.93 × 10^−4^	0.77	2.08 s

**Table 3:** Performance comparison of different methods on RADARSAT-1 dataset.

Method	ENL	ENY	RARES	Imaging Time
CSA	0.98	2.10	3.02	3.51 s
*ℓ*_1_-ITA	0.17	0.06	5.30	7.11 s
*ℓ*_1/2_-ITA	1.13	0.01	2.87	6.27 s
*ℓ*_2/3_-ITA	1.26	0.01	2.76	6.57 s
The proposed method	1.81	8.2 × 10^−3^	2.40	6.03 s

**Table 4:** Performance comparison of different methods on GaoFen-3 dataset.

Method	ENL	ENY	RARES	Imaging Time
CSA	0.22	2.35	1.22	0.50 s
*ℓ*_1_-ITA	0.63	0.71	3.52	1.53 s
*ℓ*_1/2_-ITA	6.52	0.25	1.43	0.69 s
*ℓ*_2/3_-ITA	7.65	0.17	1.33	0.74 s
The proposed method	15.92	0.098	0.97	0.67 s

## Data Availability

Data is contained within the article.
